# Using the LN34 Pan-Lyssavirus Real-Time RT-PCR Assay for Rabies Diagnosis and Rapid Genetic Typing from Formalin-Fixed Human Brain Tissue

**DOI:** 10.3390/v12010120

**Published:** 2020-01-18

**Authors:** Rene Edgar Condori, Michael Niezgoda, Griselda Lopez, Carmen Acosta Matos, Elinna Diaz Mateo, Crystal Gigante, Claire Hartloge, Altagracia Pereira Filpo, Joseph Haim, Panayampalli Subbian Satheshkumar, Brett Petersen, Ryan Wallace, Victoria Olson, Yu Li

**Affiliations:** 1 Pox and Rabies Branch, Centers for Disease Control and Prevention, Atlanta, GA 30329, USA; 2 Laboratorio de Salud Pública “Dr. Defillo”, 10105 Santo Domingo, Dominican Republic; 3 Direccion de Salud Pedernales, Pedernales 84000, Dominican Republic; 4 Centro de Prevención y Control de Enfermedades Transmitidas por Vectores y Zoonosis (CECOVEZ), 10308 Santo Domingo, Dominican Republic; 5 Oak Ridge Institute for Science and Education, Oak Ridge, TN 37830, USA; 6 Ministère de l’Agriculture, des Resources Naturelles et du Développement Rural, Department of Animal Health, HT 6110, Republic of Haiti

**Keywords:** rabies virus, lyssavirus, diagnosis, LN34 assay, formalin-fixed tissue, genetic typing

## Abstract

Human rabies post mortem diagnostic samples are often preserved in formalin. While immunohistochemistry (IHC) has been routinely used for rabies antigen detection in formalin-fixed tissue, the formalin fixation process causes nucleic acid fragmentation that may affect PCR amplification. This study reports the diagnosis of rabies in an individual from the Dominican Republic using both IHC and the LN34 pan-lyssavirus real-time RT-PCR assay on formalin-fixed brain tissue. The LN34 assay generates a 165 bp amplicon and demonstrated higher sensitivity than traditional PCR. Multiple efforts to amplify nucleic acid fragments larger than 300 bp using conventional PCR were unsuccessful, probably due to RNA fragmentation. Sequences generated from the LN34 amplicon linked the case to the rabies virus (RABV) strain circulating in the Ouest Department of Haiti to the border region between Haiti and the Dominican Republic. Direct sequencing of the LN34 amplicon allowed rapid and low-cost rabies genetic typing.

## 1. Introduction

Rabies is an acute encephalitis with an inevitably fatal outcome. Despite being a vaccine preventable disease, it has been estimated that approximately 59,000 people die annually from rabies around the world [1]. Most human rabies deaths are associated with dog bites in countries where canine rabies is endemic, while in canine rabies-free countries, terrestrial wildlife and bats pose the greatest risk for humans [2,3]. In the Americas and the Caribbean, canine rabies remains endemic in a few countries, including Haiti (HTI) and the Dominican Republic (DOM) [4]. Several social, cultural and economic factors are likely associated with a higher incidence of human rabies. Rabies continues to threaten public health in developing countries [5]. The World Health Organization (WHO) has included rabies in a list of neglected diseases worldwide. Given that rabies is incurable, once symptoms appear, appropriate wound treatment and prompt access to postexposure prophylaxis (PEP) are the bedrock for saving lives. Canine vaccination is the keystone of modern control programs to prevent and eliminate rabies [6].

*Rabies lyssavirus* is a species of single-stranded negative sense RNA viruses belonging to the *Lyssavirus* genus in the Rhabdoviridae family. RABV is able to cause disease in a wide range of carnivore and chiropteran species [7]. Humans are usually infected by a bite or scratch from a rabid animal. Human rabies infection starts with a prodromal flu-like illness, which is difficult to diagnose due to the non-specific nature of the symptoms; this phase is followed by encephalitic or paralytic manifestations at which time it is easier to recognize or suspect rabies [8,9,10]. Clinical diagnosis of rabies may be challenging if the treating physician lacks experience or there is no patient history of an animal bite; these factors can lead to inadvertently omitting rabies from the differential diagnosis [11,12]. In countries with limited resources, human rabies cases are believed to be underreported. The direct fluorescent antibody (DFA) test is currently the gold standard for postmortem rabies diagnosis recommended by WHO and the World Organization for Animal Health (OIE) [13]. The DFA test is a highly sensitive method when fresh samples are tested. However, when the condition of the sample is unsuitable, the reliability of the results may be compromised. In order to rule out rabies, preferably the brain stem and cerebellum must be included, and samples must be maintained under cold chain conditions from the point of collection to the testing location. When a patient dies of an unknown illness and rabies is not initially considered, samples collected during autopsy are commonly preserved in formalin to avoid autolysis and deterioration of structural elements of the tissue. In such cases, when rabies is suspected afterward, samples cannot be tested by the standard DFA test [14]. When only formalin-fixed tissue is available, immunohistochemistry (IHC) is the primary testing method that can be used to rule out rabies [15,16]; however, further characterization to determine the RABV strain cannot be performed. Recently, the updated OIE Manual of Diagnostic Tests and Vaccines for Terrestrial Animals included pan-lyssavirus polymerase chain reaction (PCR) assays as an acceptable test for primary diagnosis of rabies [13]. Molecular methods have been successfully applied to RABV characterization; however, amplification of viral RNA depends on the quality of RNA recovered from formalin-fixed tissue.

### 1.1. Case

On 15 July 2018, a five-year-old girl from Pedernales province in DOM arrived at the emergency room of Elias Fiallo hospital with a history of a fever (up to 38.5 °C) for five days, breathlessness, sore throat, irritability, anorexia, anxiety/panic attacks, and tachycardia. Antibiotics, antipyretics, and analgesics were prescribed with an initial diagnosis of febrile illness and moderate dehydration. A blood screening test showed neutrophilic leukocytosis (15.8 × 10^3^/mm^3^, 92.2% neutrophils) and hyperglycemia (296 mg/dL). No additional testing was recommended. On 16 July 2018, the patient showed no improvement in response to treatment of symptoms, and her health worsened, presenting with alteration of behavior, hyperactivity, anxiety/panic attacks, and hallucinations. At this time, her parent requested she be transferred to a tertiary care hospital in Santo Domingo. The patient was referred to a pediatric psychology department with alteration of consciousness and rheumatic fever diagnosis. The father took the patient to Santo Domingo by public transportation. While in route she began vomiting and died while in the process of being admitted to the Hospital Taiwan in Azua province. Febrile illness and unknown history of animal exposure was the initial clinical diagnosis after the patient was deceased. Brain tissue was collected after authorization by the district attorney and preserved in formalin for pathology examination. A diagnosis of rabies was suspected based on the patient’s clinical symptoms. The DOM National Laboratory of Public Health “Dr. Defillo” submitted formalin-fixed brain cortex tissue to the rabies laboratory at the Centers for Disease Control and Prevention (CDC) in the United States. The sections of the brain required to rule out rabies (brain stem and cerebellum) were not submitted. RABV antigen was detected in the formalin-fixed tissue on August 3 by IHC. Specific RABV nucleic acid was also detected from different sections of the formalin-fixed brain tissue by the LN34 real-time RT-PCR assay.

### 1.2. Public Health Investigation

Once the diagnosis of rabies was confirmed by the CDC and communicated to the DOM National Laboratory of Public Health, an outbreak response team was activated, consisting of personnel from the Dirección de Salud Pedernales and the Centro de Prevención y Control de Enfermedades Transmitidas por Vectores y Zoonosis (CECOVEZ). Rabies prevention and control activities were implemented with a full investigation and evaluation of the case. After several interviews with the parents, family, and neighbors, it was determined that the patient was bitten by a dog of unknown origin or whereabouts approximately four months before the onset of symptoms. Due to lack of knowledge about rabies, her guardians (grandparents) did not seek medical care and PEP was not administered. Instead, acetaminophen was given without medical prescription, and the local witch doctor of the community “tres charcos” in the Oviedo municipality looked after the patient’s initial symptoms, giving a beverage known locally as “tomo”. An extensive review of emergency rooms, private clinics, and community health post records were conducted, identifying 231 cases of dog bites in the period from January to August 2018; PEP administration was recommended when appropriate. During the course of the field investigation, PEP was administered to the parents, close relatives, and healthcare personnel (*n* = 51) who had close contact with the patient and/or the rabid dog.

## 2. Materials and Methods

### 2.1. Samples

Post mortem human formalin-fixed brain tissue (A18-2173) used in this study was collected in DOM during autopsy; an unrecognizable area of the brain cortex was submitted for testing. Additional archived animal samples obtained during routine rabies field surveillance in DOM and HTI were included.

### 2.2. Ethics Statement

All samples used in this study were submitted to the CDC for rabies diagnosis; this study did not require ethics committee approval.

### 2.3. Immunohistochemistry (IHC)

Upon receipt at the CDC laboratory, the brain tissue of A18-2173 was placed into fresh 10% buffered formalin for 24 to 48 h, then prepared for RABV antigen detection by IHC as described previously [17,18]. Briefly, three areas of the brain cortex were collected; sections of 3 to 5 mm were cut from the formalin-fixed brain, placed into cassettes, processed in a tissue processor to formalin-fixed paraffin-embedded blocks, and finally sectioned on a microtome to formalin-fixed paraffin embedded slides. Primary rabbit polyclonal antibodies, mouse serum, and secondary biotinylated goat anti-rabbit antibody were used to perform the IHC.

### 2.4. LN34 Real-Time RT-PCR Assay

To confirm RABV infection, nucleic acid was extracted from three areas of the brain tissue wherein the IHC detected high concentrations of RABV antigen, three areas with low concentration, and two from gray and white matter areas that were untested by IHC. Approximately 0.01 g of tissue was dissociated mechanically using ceramic beads in 360 µL of lysis buffer with 40 µL proteinase K (DNeasy Blood and Tissue Kit—Qiagen, Hilden, Germany) and incubated overnight at 55 °C. Then 100 µL of homogenized tissue was mixed with 400 µL of Trizol; the RNA extraction and the LN34 assay were performed as described in detail previously [19,20].

### 2.5. Genetic Typing Analysis

To determine the RABV variant, the amplicons produced by the LN34 assay were purified using a MinElute PCR Purification kit (Qiagen, Hilden, Germany), and direct sequencing was performed using BigDye Terminator v1.1 on the ABI 3730 DNA Analyzer (Thermo Fisher Scientific, Waltham, MA, USA). Both the sequencing reaction and sequencing run were shortened due to the short DNA fragment size of the LN34 amplicon. To compare the sequence of RABV isolate A18-2173 with previous isolates across DOM and HTI, the LN34 amplicons from archived isolates were sequenced, including one sample from a previous human rabies case that occurred in the province of Santiago in DOM and 17 samples from rabid domestic animals (Table 1). To perform the phylogenetic analysis, sequences were assembled and edited in Bioedit [21]. LN34 primer sequences were removed from the amplicon, and 115 bp sequences were used for the comparison. Representative sequences from GenBank were retrieved and multiple alignment was performed by ClustalW. Reconstruction of the phylogenetic tree was performed by the Bayesian Markov chain Monte Carlo (MCMC) method using BEAST v1.8.4 software [22]. In addition, traditional RT-PCR and hemi-nested PCR were performed using 1066 deg F, 1087Sdeg, and 304^+^ primers [23].

## 3. Results

### 3.1. Immunohistochemistry (IHC)

**Figure 1:**
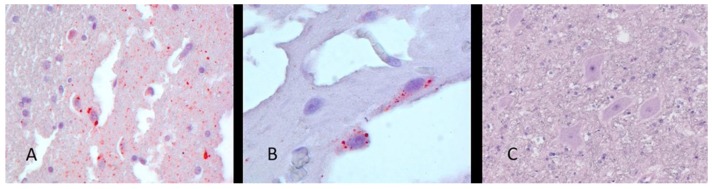
Immunohistochemical (IHC) staining for RABV antigen in fixed brain tissue from the Dominican Republic (DOM) patient (A18-2173). (**A**) RABV antigen detection at 400× magnification. (**B**) Neuronal cytoplasmic inclusions at 630× magnification. (**C**) Negative control brain at 400× magnification. Streptavidin–biotin complex staining method using rabbit antibodies against RABV nucleoprotein, signal development with AEC chromogen (magna red), and Gills hematoxylin counterstain.

Brain sections from A18-2173 were collected on three slides and tested by the IHC test for rabies antigen. All slides contained RABV antigen. Antigen distribution was largely sparse throughout the tissue sections. Some areas contained foci of higher antigen concentrations (visible as red stained foci, equal to a 2+ distribution with 4+ intensity) (Figure 1). Based on the IHC findings, representative areas of the formalin-fixed brain tissue were selected for molecular testing.

### 3.2. LN34 Assay—Rabies Diagnostics and Genetic Typing

Three areas of brain tissue where IHC detected a high concentration of RABV antigen and three with low antigen distribution tested positive by the LN34 assay. Specific RABV nucleic acid was not detected in one of two samples from the gray and white matter areas that was not tested by IHC. The relative quantities of RABV RNA, measured based on the LN34 cycle threshold (Ct) value, ranged from 26.1 to 35 for seven samples. The presence of host RNA was determined by a real-time RT-PCR assay that detects housekeeping gene β-actin; Ct values ranged from 22.4 to 27.6 (Table 2). Traditional RT-PCR and hemi-nested PCR consistently failed to amplify a larger portion of nucleoprotein gene (370–390 bp). The LN34 amplicon was a 164 base pair (bp) DNA fragment covering at the 3′ end leader region of the RABV genome and a portion of nucleoprotein gene. The short LN34 amplicon allowed a quicker sequencing reaction and sequencing run, so genetic typing results were generated within 3 h after completing the LN34 assay.

**Figure 2:**
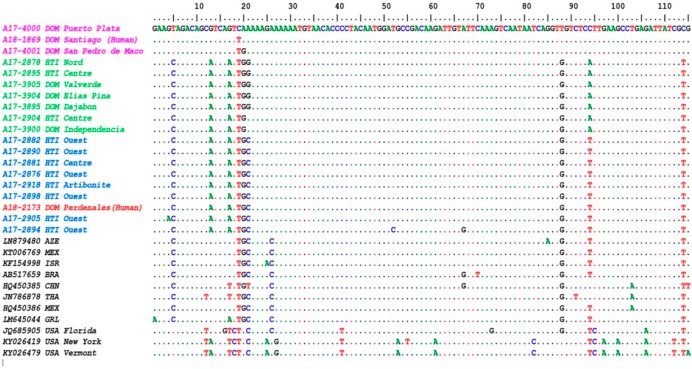
Sequence alignment (115 bp) from LN34 amplicons link the human case A18-2173 (labelled in red) to the RABV strain (blue) circulating mainly in the Ouest Department of Haiti (HTI). Three groups of RABV were identified based on the sequence alignment: HTI contains two groups (blue and green) and DOM has three groups (blue, green, and purple).

Both the forward and reverse primers of the LN34 assay contain degenerate bases. To avoid potential errors during sequence comparisons, the primer sequences were removed from the final sequences; the remaining LN34 sequence was 115 bp long and was compared with LN34 sequences of archived isolates from DOM and HTI (Table 1) and sequences deposited in GenBank. Among 19 samples included in this study, there were three distinct sequence patterns labelled purple, green, and blue in the sequence alignment in Figure 2. The LN34 amplicon sequence from human case A18-2173 (in red) from Pedernales was identical to 6 sequences from samples collected in the Quest Department of HTI (sample ID in blue), a town (Artibonite) on the east coast (A17_2918), and the Centre Department (A17-2881). Among this cluster of samples, additional nucleotide changes were present in two cases (A17-2905 and A17-2894) from the Quest Department.

The sequences that included samples originating in areas in northern HTI and the region surrounding the border of HTI and DOM (sample ID in green) contained nucleotide substitutions in position 21 and 94 of the alignment (Figure 2). A previous human rabies case from DOM (A18_1869) and two other sequences from samples collected in the northern and southeastern provinces of the DOM (labelled in purple) contained up to eight nucleotide substitutions compared to two clusters of rabies viruses above. The RABV isolates in HTI and DOM contained the specific single-nucleotide polymorphism (SNPs) patterns of RABV Cosmopolitan variants. For sequences containing complicated SNP patterns, phylogenetic analysis has advantages in understanding sample relationships.

### 3.3. Phylogenetic Analysis Using LN34 Amplicon Sequences

**Figure 3:**
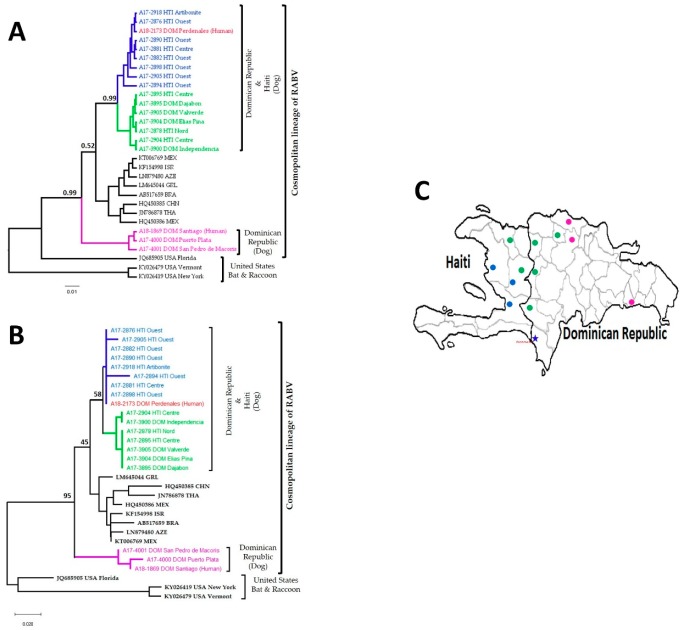
Molecular phylogenetic analysis using 115 bp sequences generated from LN34 amplicons. (**A**) The Bayesian evolutionary analysis sampling tree (BEAST) showed that the human rabies case (A18_2173) grouped with samples from HTI. The major nodes are labelled with the posterior probabilities. * Posterior values > 0.5 are shown at each node. (**B**) The neighbor joining tree agreed with the BEAST tree for the major clades and showed strains with identical sequences. The selected nodes are labelled with bootstrap values. (**C**) The geographic location of samples from HTI and DOM. The blue star is the location where the patient was infected.

Two phylogenetic analysis methods were performed using the LN34 amplicon sequences; the phylogenies from both the Bayesian evolutionary analysis sampling tree (BEAST) (Figure 3A) and the neighbor joining (NJ) tree (Figure 3B) showed that the RABV in DOM and HTI belong to the Cosmopolitan variant type, divided into three subclades. The human rabies case from Pedernales (A18_2173, red) was closely related to the RABV clade circulating in the Ouest Department of HTI labelled in blue (Figure 3); results were confirmed using direct sequence comparison (Figure 2). The RABVs clustered in the green clade were broadly dispersed along the mountain range, while the RABVs in the purple clade that grouped samples collected in the northern and southeastern provinces of DOM were clearly different from the blue and green clades that were established in the border regions (Figure 3C). Both the posterior scores and bootstrap values showed similar relative values in the clustering of the three subclades, but the BEAST tree and NJ tree had slightly different topologies and the NJ tree had clear indications of the samples with identical sequences.

## 4. Discussion

Dogs are the primary source of rabies disease in many endemic countries, causing up to 99% of human RABV infections, with at least 40% of these cases in children under 15 years [6,24]. Clinical manifestations in human rabies can be very variable, from flu-like symptoms to full blown encephalitis, and multiple laboratory methods are needed to accurately rule out rabies. In impoverished rural endemic areas, where most rabies cases occur, timely access to laboratory testing may be constrained. In this situation, the observation of clinical signs and symptoms is still used to diagnose rabies. In such settings, due to a variable clinical manifestations, laboratory assistance may be required [6,25,26]. The DFA test is the gold standard for rabies diagnosis; the method requires an expensive fluorescence microscope, which is often only available at the national level. The accuracy of the DFA test is also determined by the quality of the sample and relies on testing different sections of the brain stem and cerebellum. The antigenic typing requires multiple RABV species- or strain-specific antibodies, and additional experiences in testing and analyzing testing results, and is difficult to be used for the rabies surveillance in rabies endemic areas. In remote areas where access to the cold chain is limited, the sample may be preserved in formalin. Previous studies determined that the fluorescent antibody method is not reliable for testing formalin fixed samples because its reproducibility is limited [27,28]. In these circumstances, the first tool to rule out rabies is IHC.

The formalin-fixed tissue outlined in this study tested positive by IHC and the results were promptly reported to implement appropriate measures and prevent additional human cases; however, the RABV strain characterization was not performed due to limits of IHC to distinguish RABV strains. A new molecular approach, the LN34 real-time RT-PCR assay, has recently demonstrated the ability to amplify and detect a 165 bp fragment of the RABV genome in formalin-fixed tissues [20]. We successfully generated RABV amplicons using the LN34 assay in seven out of eight samples, with Ct values ranging from 26.1 to 35. Six samples that tested positive by IHC also tested positive by LN34 assay (100% diagnostic sensitivity). In addition, we included two samples from cortex areas (white and gray matter) of the brain that were not tested by IHC; a sample from the gray matter of the cortex tested negative. This result may be directly associated with the RABV infection in the brain tissue. It is known that RABV does not infect the brain uniformly; therefore, testing multiple areas of the brain is advantageous [29]. To accurately rule out rabies, a full cross section of the brain stem is the most reliable part of the brain; other parts, like the hippocampus, cerebellum, or cerebrum can even give false negative results [15,29,30]. Although brain stem was unavailable in this case, the LN34 assay demonstrated its capacity to amplify RABV nucleic acid in formalin-fixed samples providing an alternative tool to confirm RABV infection in fixed clinical samples. Multiple efforts to generate amplicons larger than 300 bp by conventional PCR failed, probably due to nucleic acid fragmentation during tissue fixation, which can limit the obtaining of fragments to between 100 and 200 bp in length [31,32,33]. The LN34 assay can be implemented in any laboratory with qPCR platforms and improve the diagnosis of fixed brain tissues. For the laboratories without sequencing capacities, LN34 amplicons can be shipped and sequenced at a regional center.

RABV amplicons generated by the LN34 assay were purified and sequenced by the Sanger method. Then, 115 bp long sequences were used for comparison and phylogenetic analysis; archived isolates and reference sequences retrieved from GenBank were also included. A direct sequence comparison using multiple references can provide quick and straightforward results if the sequences of the clinical samples have identical or minimal sequence changes from reference sequences. In this report, the LN34 amplicon sequences from the patient matched perfectly with six canine RABV samples in the surrounding area. LN34 amplicon sequences are highly conserved; a few or even a single nucleotide change can be significant in separate RABV variants or clusters. If aligned sequences demonstrated complicated mutation patterns, the phylogenetic analysis is more reliable to understand the relationships of samples used in the comparisons. The phylogenetic analysis methods revealed a distinct monophyletic clade containing all HTI isolates and those isolates from DOM collected in the border range of the central mountains. Isolates from the eastern regions of DOM were grouped separately within the larger canine cosmopolitan lineage (Figure 3). Our findings indicate the human case from Pedernales was infected with a RABV strain commonly found in domestic dogs.

HTI has the highest burden of canine rabies in the western hemisphere [34]; however, little is known about the RABV reservoirs across the island. Using the short length of the LN34 amplicons, a direct sequence comparison and phylogenetic analysis determined at least three distinct canine rabies subclades across the island. The human case from Pedernales was clustered within the clade established mainly in the Ouest Department of HTI, which includes the capital Port-au-Prince. Members of this clade were dispersed in the coastal regions of HTI, including the coastal province of Pedernales in DOM. To understand the distribution of this clade, more sequences from neighboring regions are certainly needed. The phylogenetic tree clearly distinguished a clade established in DOM. This clade was widespread on the east side of the island; however, distribution of this clade in the central part of the island including the mountain is still unknown. Notably, the phylogenetic tree revealed a circulation of a third clade across the north mountain range border shared between both countries (clade in green, Figure 2 and Figure 3). Members of this clade were also found in the Nord Department in HTI and Valverde province in DOM, but it is still unclear if these cases are related geographic translocation or widely spread in both countries.

LN34 amplicon sequences were correct in determining that the purple subclades of DOM (Figure 3) were the most diverged among the RABV strains circulated in HAI and DOM; however, the topology has differences comparing the tree generated from the full N gene (manuscript in preparation). Although there are variations in the phylogenetic analysis using short sequences, LN34 amplicon sequencing produces rapid genetic typing results and useful phylogenetic information. For the comprehensive phylogenetic analysis of RABV samples, longer sequences are preferred. In this reported case, only LN34 amplicon sequences can be generated using the traditional Sanger sequencing method, a standard method for the sequencing of rabies viral genes. In the future, the next-generation sequencing technology can be an alternative method to sequence the samples of fixed or highly fragmented RABV samples. Obtaining phylogenetic information from formalin fixed samples is helpful to determine the relationship between viruses from different geographic locations [35]. Our results contribute to the knowledge of RABV diversity in HTI and DOM and to understanding the molecular epidemiology of rabies across the island.

## 5. Conclusions

The improved sensitivity of the LN34 assay has been demonstrated using multiple types of tissues including fresh, frozen, and fixed tissues and tissues in poor condition [19,20]. In this reported case, the LN34 assay was able to detect viral nucleic acid in a formalin-fixed clinical sample, and the LN34 amplicon sequencing provided genetic typing information for the cases using fragmented RNA. LN34 amplicon sequence comparison and phylogenetic analysis determined the rabies variant in this human case and showed the epidemiologic links of the cases as well the genetic diversity of rabies across the island. This case report indicated that implementation of the LN34 assay can improve rabies diagnosis and surveillance. This approach provides practical ways to add rabies diagnostics and surveillance capacities globally, which is critical to achieve the goal of canine rabies elimination by the end of the next decade.

## Figures and Tables

**Table 1:** Isolates of rabies virus (RABV) used in the phylogenetic analysis.

ID	Country	Province	Host	Year
A17-3895	Dominican Republic	Dajabón	Dog	2017
A17-3900	Dominican Republic	Independencia	Dog	2017
A17-3904	Dominican Republic	Elías Piña	Dog	2017
A17-3905	Dominican Republic	Valverde	Cat	2017
A17-4000	Dominican Republic	Puerto Plata	Dog	2017
A17-4001	Dominican Republic	San Pedro de Macorís	Dog	2017
A18-1869	Dominican Republic	Santiago	Human	2018
A18-2173	Dominican Republic	Perdenales	Human	2018
A17-2904	Haiti	Centre/Mirebalais	Dog	2016
A17-2905	Haiti	Ouest/Tabarre	Dog	2016
A17-2918	Haiti	Artibonite/Saint-Marc	Dog	2016
A17-2876	Haiti	Ouest/Petionville	Dog	2017
A17-2878	Haiti	Nord/Cap-Haitien	Dog	2017
A17-2881	Haiti	Centre/Saut-D-eau	Dog	2017
A17-2882	Haiti	Ouest/Port-Au-Prince	Dog	2017
A17-2890	Haiti	Ouest/Croix-Des-Bouqets	Dog	2017
A17-2894	Haiti	Ouest/Petit-Goave	Dog	2017
A17-2895	Haiti	Centre/Thomassique	Dog	2017
A17-2898	Haiti	Ouest/Croix-Des-Bouqets	Dog	2017

**Table 2:** Comparison of IHC and the LN34 Taqman Real-Time RT-PCR assay using multiple sections of brain tissues of A18-2173.

IHC RABV Distribution/Intensity	Real Time RT-PCR		
	LN34 Ct	β-Actin * Ct	Result
2+/4+	30.7	24.5	Positive
2+/4+	26.1	27.6	Positive
2+/4+	26.9	22.4	Positive
<2+/4+	34.5	24.1	Positive
<2+/4+	35	24.8	Positive
<2+/4+	32.5	26	Positive
Not tested white matter	30.7	24	Positive
Not tested gray matter	Undetected	25.5	Negative

* qPCR assay measures host RNA level.
